# Influence of Low-Pressure RF Plasma Treatment on Aramid Yarns Properties

**DOI:** 10.3390/molecules25153476

**Published:** 2020-07-30

**Authors:** Alicja Nejman, Irena Kamińska, Izabela Jasińska, Grzegorz Celichowski, Małgorzata Cieślak

**Affiliations:** 1 ŁUKASIEWICZ–Textile Research Institute, Department of Chemical Textiles Technologies, 5/15 Brzezinska Street, 92-103 Lodz, Poland; ikaminska@iw.lodz.pl (I.K.); ijasinska@iw.lodz.pl (I.J.); cieslakm@iw.lodz.pl (M.C.); 2 Department of Materials Technology and Chemistry, University of Lodz, Pomorska Street 163, 90-236 Lodz, Poland; grzegorz.celichowski@chemia.uni.lodz.pl

**Keywords:** meta-aramid, para-aramid, low-pressure RF plasma treatment, surface free energy, differential scanning calorimetry, thermogravimetric analysis, FTIR spectroscopy, specific strength

## Abstract

The aim of the study was to modify the surface free energy (SFE) of meta- (mAr) and para-aramid (pAr) yarns by their activation in low-pressure air radio frequency (RF) (40 kHz) plasma and assessment of its impact on the properties of the yarns. After 10 and 90 min of activation, the SFE value increased, respectively, by 14% and 37% for mAr, and by 10% and 37% for pAr. The value of the polar component increased, respectively by 22% and 57% for mAr and 20% and 62% for pAr. The value of the dispersion component for mAr and pAr increased respectively by 9% and 25%. The weight loss decreased from 49% to 46% for mAr and 62% to 50% for pAr after 90 min of activation. After 90 min, the specific strength for mAr did not change and for pAr it decreased by 40%. For both yarns, the 10 min activation in plasma is sufficient to prepare their surface for planned nanomodification.

## 1. Introduction

Aromatic polyamides, meta- and para-aramid, due to their properties, such as thermal and mechanical resistance [1,2], high thermal conductivity, and hydrophobicity, are widely used in many industries, such as aviation, ballistics, or special clothing production. To give them new functional properties and expand their area of application, research is carried out into their modification. The non-polar nature of the aramid fibers and the smooth surface cause difficulties in application and chemical binding with the modifier. Aramid surface treatment techniques, such as mechanical, chemical, and plasma treatment are used to improve the adhesion of composite products [3,4,5,6,7,8,9]. Since the diameter of the fiber is usually several micrometers, the use of mechanical methods significantly deteriorates its properties. In recent years, problems related to environmental pollution have also limited the wide industrial applications of chemical surface treatment [3]. That is why plasma treatment, especially the most commonly used air plasma, as an environmentally friendly technique, has become an important process of surface modification [4,5,6,7,8,9]. Plasma for a wide range of polymers allows the formation of oxygen functional groups on the surface [6,7,10]. During this kind of treatment, two processes occur simultaneously: etching of the polymer surface [8,11,12,13] by the reaction of free radial species created from water vapor together with atomic oxygen with carbon atoms and the creation of active polar functional groups [5]. Plasma processing involves modification of only the outermost atomic layers without affecting their mechanical properties [4], increase the surface free energy and its dispersive and especially polar component [14,15,16]. An important advantage of plasma treatment is changing the topography together with creating active polar functional groups [7,8,9,14].

Such treatment is used for various types of fibers [17,18]. In our previous work, we activated woven fabrics: meta and para-aramid, using corona discharges with different doses of activation energy [15]. In this work, we wanted to investigate how activation in the plasma will affect the properties of stable structures of yarn. To what extent they will be modified under the influence of plasma and how it will affect its properties in terms of its further functionalization.

The aim of the study was to modify the free surface energy and its polar and dispersive component of meta-aramid (mAr) and para-aramid (pAr) yarns after low-pressure air radio frequency (RF) plasma treatment after 10, 30, 60, and 90 min and to assess the impact of process parameters on changes in thermal and mechanical properties. Based on the obtained results, it will be possible to select the appropriate modification parameters for further functionalization and proper processing of the modified yarn into textile structures.

In this work, the analysis of surface topography and the elemental composition was made using Scanning Electron Microscopy/Energy Dispersive Spectroscopy (SEM/EDS). To assess the changes on the fibers surfaces the Fourier Transform Infrared Spectroscopy (FTIR) was made. The changes in surface properties: the surface free energy and its polar and dispersive component were determined using goniometric analysis. The thermal properties were determined using Differential Scanning Calorimetry (DSC) and Thermogravimetry/Derivative Thermogravimetry (TG/DTG). The mechanical properties were studied.

## 2. Results and Discussion

### 2.1. SEM/EDS Analysis

**Figure 1:**
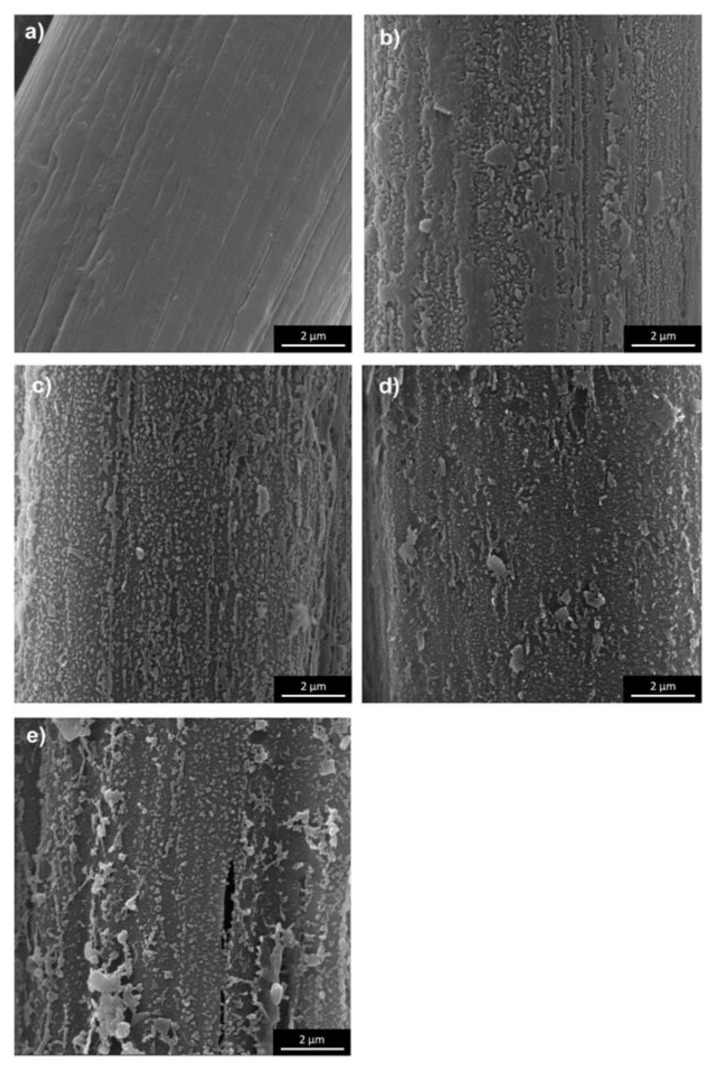
SEM images of the fiber surface of: (**a**) reference, (**b**) 10 min, (**c**) 30 min, (**d**) 60 min, (**e**) 90 min plasma-treated meta-aramid (mAr) yarn (magnification 20000×).

**Figure 2:**
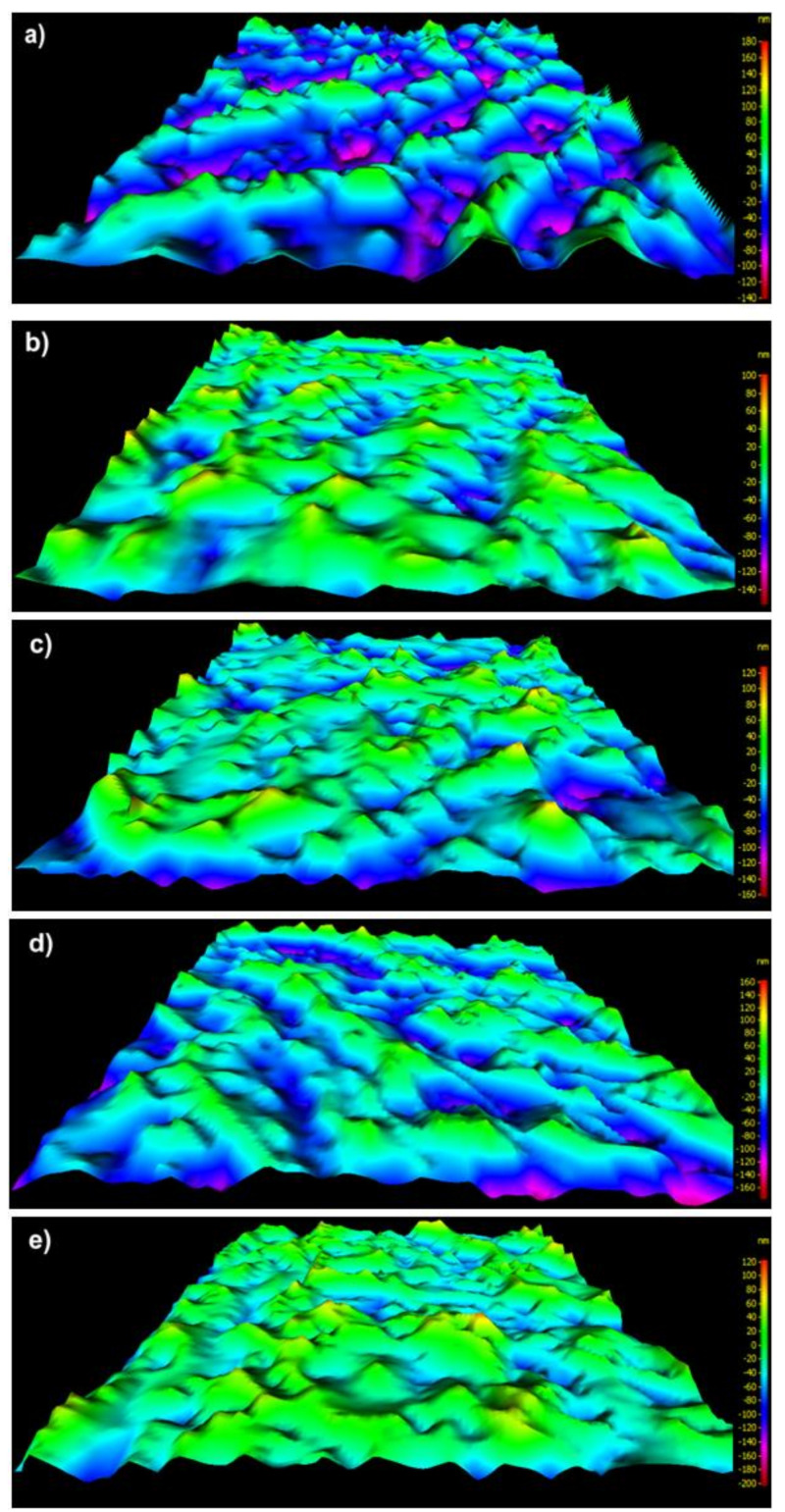
The 3D SEM images of the fiber surface of: (**a**) reference, (**b**) 10 min, (**c**) 30 min, (**d**) 60 min, (**e**) 90 min plasma-treated mAr yarn.

**Figure 3:**
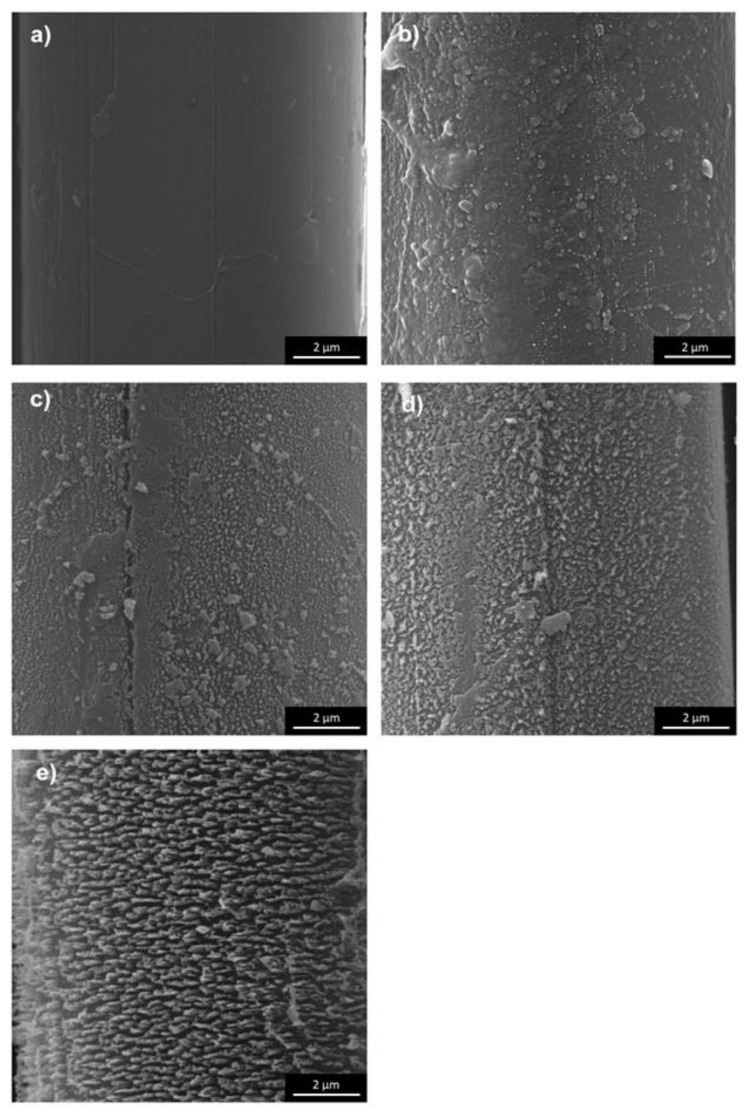
SEM images of the fiber surface of: (**a**) reference, (**b**) 10 min, (**c**) 30 min, (**d**) 60 min, (**e**) 90 min plasma-treated pAr yarn (magnification 20000×).

**Figure 4:**
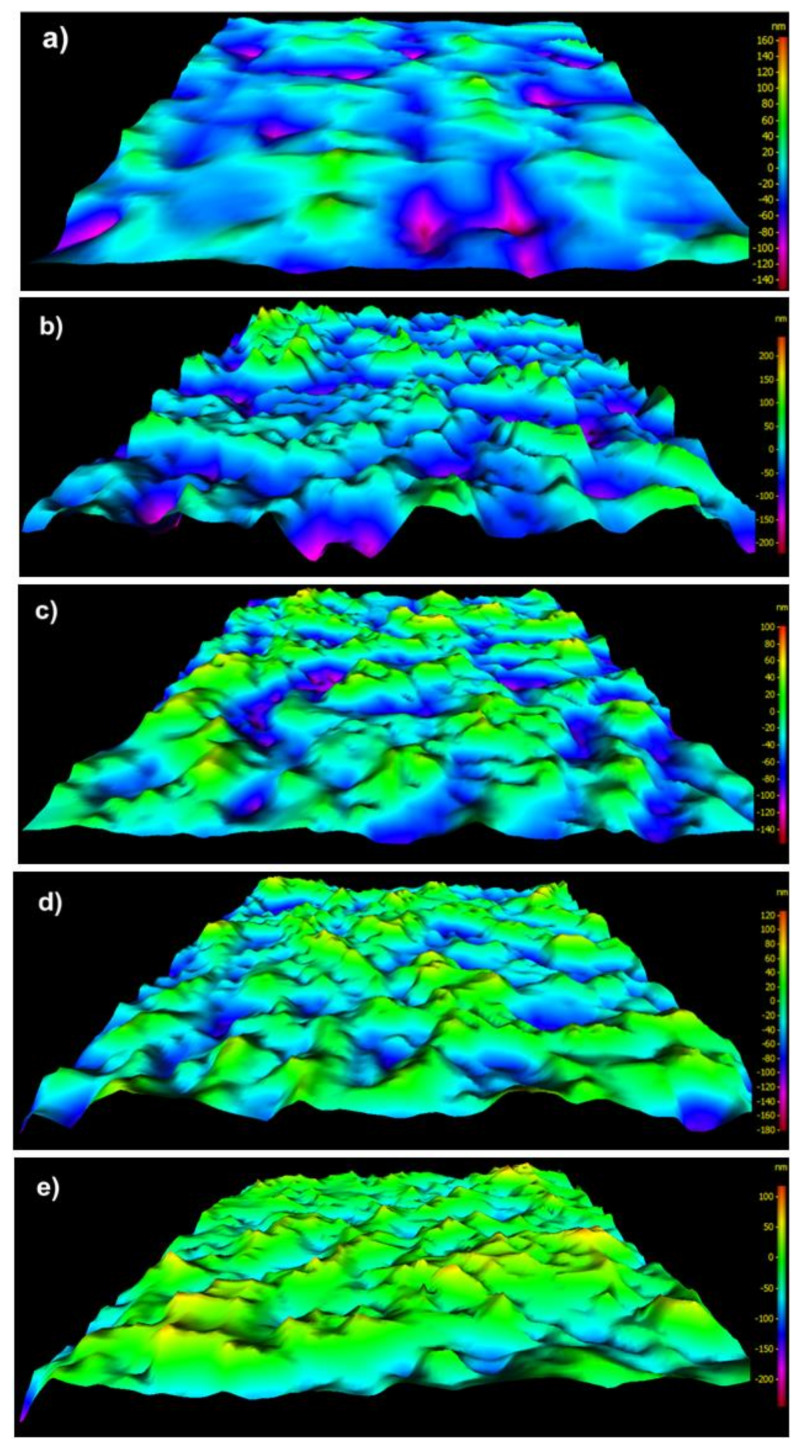
The 3D SEM images of the fiber surface of: (**a**) reference, (**b**) 10 min, (**c**) 30 min, (**d**) 60 min, (**e**) 90 min plasma-treated pAr yarn.

The fibers surface of unmodified meta-aramid (mAr/0) and para-aramid (pAr/0) yarns are smooth with visible longitudinal cracks and fibrils [6,15]. Activation in the plasma caused changes in surface topography, increased unevenness and roughness, as evidenced by SEM three-dimensional (3D) images and stereometric parameters (Figure 1, Figure 2, Figure 3 and Figure 4). The parameter of the surface topography; the average height of the selected area (S_a_), the maximum depth of the valley of the selected area (S_v_), and the maximum height of the selected surface area (S_z_) for mAr/0 yarn are 23 nm, 133 nm, and 245 nm, respectively. The surface area development factor (S_dr_) is 18%. An increase in the value of S_a_, S_v_ by 50%, S_z_ by 45%, and S_dr_ by about 7% is observed for mAr yarn after 60 min of plasma activation (mAr/60) (Figure 2d). After 90 min of activation, the S_v_ value increased to 203nm, and the values of S_a_, S_z_ and S_dr_ decreased by 17%, 5% and 14%, respectively, in relation to mAr/60. It can be caused by too much energy and removal of the top layer on which there were areas with the developed surface. This is evidenced by the protruding fragments on the fiber surface (Figure 2e).

The values of S_a_, S_v_, and S_z_ of pAr/0 yarn are 23 nm, 152 nm, and 317 nm, respectively (Figure 4a). Longitudinal cracks on the surface are deeper than for mAr/0. The S_dr_ for pAr/0 is 12% and is 30% lower compared to mAr/0. After 10 min of activation, there was an increase in S_a_ value of about 26% compared to pAr/0 (Figure 4b). After 30 and 60 min (Figure 4c–d), the S_a_ value does not change further, but after 90 min decreases by 10% compared to pAr/60 (Figure 4e). The value of S_v_ after 10 and 30 min of activation is lower by 10% and 5%, respectively, and after 60 and 90 min is higher by 30% and 60%, respectively, in relation to pAr/0. The S_z_ value after 10 min and 30 min activation is lower by 12% and 18%, respectively, compared to pAr/0. After 60 min, an increase in S_z_ value by about 18% was recorded, relative to pAr/30. The highest value of S_z_ is observed after 90 min of activation and it is 364 nm. The S_dr_ increases by approximately 133%, 116%, and 141% for pAr/10, pAr/30, and pAr/60 yarns, respectively, compared to pAr/0. For pAr/90, the S_dr_ value decreases by 28% compared to pAr/60.

It can be seen that there is a clear difference in the surface morphology of unmodified yarns and both types of aramid fibers after plasma treatment. On the relatively smooth surface covered with longitudinal cavities and microfibrils, no protruding fragments are visible. Still, on the rough surface of aramid fibers after activation, numerous cavities, and fiber fragments are resulting from plasma etching. Plasma treatment causes the polymer degradation due to ion, electron, and free radical bombardment. The results of surface roughness analysis are shown in Figure 2 and Figure 4. The trends of surface roughness changes were in line with the trends visible on the SEM and 3D SEM images. As the plasma treatment time increased, more and larger fragments were evenly distributed over a much rougher surface. When the plasma treatment time increased to 90 min, the surface roughness of both types of aramid decreased, which can be attributed to the continuous etching or ablation process.

This corresponds with Wang et al. [19] results who modified para-aramid yarn using plasma-induced vapor phase graft polymerization (PIVPGP) in the atmosphere of O_2_, N_2_, and Ar (100 W, 5, 10, 15, 20 min). An increase in porosity and furrows on the surface of the meta-aramid fibers treated by the microwave plasma (200 W) was observed by Ji et al. [14]. Influence an atmospheric pressure plasma of Diffuse Surface Coplanar Barrier Discharge (DSCBD) on changes of roughness for meta- and para-aramid (300 W, 30 sec, 2 min, and 5 min) studied Stepankova et al. [6]. They found an increase in the roughness of the meta-aramid fibers, and no changes for the para-aramid fibers, as in the study of Biro et al. (50–60 W,200 s) [10]. Teams [4,7,20] using plasma: NH_3_, O_2_ and H_2_O (30 W, 10 min) [4], atmospheric dielectric barrier discharge (DBD) plasma (143.5 W, 12 s) [7], atmospheric pressure plasma and He/air mixture as working gas (5, 30, 60 s) [20] found no change in the topography of para-aramid fibers surface. Poncil et al. [21] observed surface smoothing and fading of fibrils on the surface of para-aramid yarn fibers, using atmospheric plasma (60 W, 3 min) and nitrogen as a working gas. In turn, Sheu et al. [22] modifying para-aramid yarn with NH_3_, O_2_, H_2_O plasma (100 W, 10 min) noted an increase in roughness and unevenness on the surface for all three types of gases. Biswas et al. [9] observed an increase in the roughness of para-aramid fibers with time of activation at atmospheric pressure plasma (4 kW, 30 s, 1 min, 2 min, 3 min). Brown et al. [23], by treating the para-aramid fabric with NH_3_ plasma (100 W from 0.25–20 min) observed an increase in surface fibrillation. Chen et al. [24] investigated the effect of atmospheric pressure plasma (720 W) on surface topography changes of para-aramid 3D fabric subsequent layers. They noticed a significant increase in the surface roughness mainly on the fibers surfaceof the first layer of 3D fabric and a decrease in the roughness of deeper fabric layers. Chen et al. [13], by examining the effect of oxygen plasma (200 W, 5, 10, 15, 20 min) found an increase in roughness of para-aramid yarn with the activation time. Gu et al. [11] observed an increase in the unevenness of the surface of para-aramid fibers after treating with a dielectric barrier discharge (DBD) argon plasma (300 W, 60 s). Lange et al. [12] found an increase in the porosity of the surface of para-aramid fibers using atmospheric plasma (400 W, 0.1 s) and air and nitrogen. An increase in fibrillation on the surface of para-aramid fabric was observed by Guo et al. [25] after air plasma (50 W, 25 min), similarly as Jia et al. [8],after treating para-aramid fibers with DBD atmospheric plasma (143.5 W, 18 s).

SEM/EDS analysis of mAr/0 and pAr/0 yarns shows the presence of C, N, and O and trace amounts of Na and S (only for pAr) (Table 1). The presence of sodium and sulfur is due to the aramid fibers producing technology. Form Ar the Na content indicate from the reaction of two comonomers in tetrahydrofuran. The slurry of an oligomer is formed which has been contacted with sodium carbonate and the polymer is formed [26]. During plasma treatment, organic preparation is removed from the surface of the fibers, hence the reduction of C and N content. The O content increase with the activation time. The Na content amounts 0.1 wt.% and does not change after 30 min. For a longer time no Na content is observed what can be because of the tearing off fiber fragments what is observed on SEM and 3D SEM images. The pAris produced from the condensation of 1,4-diaminobenzene and terephthaloyl chloride. The formed pAr is immersed in sulfuric acid and then sodium hydroxide is added to neutralize it. The product of neutralization is sodium sulfate [26]. As for mAr yarn, plasma treatment causes the removal of organic preparation and the reduction of carbon and nitrogen content. Sodium sulfate remains on the surface. Hence, the increase in oxygen content and a slight increase in Na (from 0.5 wt.% to 0.8 wt.%) and S (from 0.5 wt.% to 0.8 wt.%) content after 60 min of plasma treatment. For 90 min activation time the decrease of Na and S is observed, which proves the detachment of macromolecular fragments, what is visible on the SEM and 3D SEM images. To verify that changes in the percentages of elements after plasma modification are statistically significant, an analysis of significant differences was performed using the t-Student’s test. Experimental values of t (Table 2) are calculated for p = 0.05 and t (0.05, 3) = 2.78 (t values from statistical tables) for the mean values of percentage C, N, O (Table 1).

The oxygen content on the surface of both fibers types increases with increasing plasma activation time.

In the case of meta-aramid yarn, the largest and statistically significant increase in O content is observed after 60 and 90 min of activation (Table 2) by 1.4 wt.% and 1.6 wt.%, respectively in relation to mAr/0. Activation causes a slight decrease in the content of C and N. These changes are not statistically significant. After 10 and 30 min, the Na content has not changed, and after 60 min there is no Na observed. Similarly, Stepankova et al. [6] found an increase in O content, a decrease in C content and no change in the N content for meta-aramid fibers.

For pAr yarn, a statistically significant increase in O content is observed for all activation times and the highest of 2.4 wt.% is for 90 min. The increase in time causes a statistically significant reduction in carbon content after 10, 30, and 60 min. The reduction of N content is statistically significant after 90 min. Much higher increase of O content and decrease of C and N content observed Jia et al. [7], Wu et al. [27], and Wang et al. [16] in comparison to our research. In addition, Lange et al. [12] observed high increase in O content. Contrary to us, Jia et al. [28] shown a decrease in O and an increase in C content. In turn, Gu et al. [11] noted a decrease in O, C and N content. Biswas et al. [9] found a slight decrease in the O and N content and increase in the C content. Changes in the percentage of elements on the surface of aramid textile materials result from the use of different types of plasma, plasma treatment conditions, and the residue of spinning preparations.

For pAr yarn, the O content before and after plasma modification is higher than for mAr yarn. These differences result from the molecular chain configuration and substitution of aromatic groups. Para-aramid consists of long molecular chains that are highly oriented and show strong intermolecular bonds in the para position. The hydrogen bonds formed from the para-substituted skeleton connect adjacent chains to form stacked sheets that curl into “microfibrils” and combine to form a fiber. This hierarchical structure allows the para-aramid to have a more even crystal structure. In the meta-aramid, the amine functional group is located in the meta orientation on the phenyl ring in the monomer. This slight difference in the molecular structure changes the optimal binding angles of phenyl-nitrogen and phenyl-carbon, causing a “crumpled” chain structure that cannot crystallize into stacked sheets. This causes it to take on a disordered structure with randomly arranged polymer chains, which results in lower crystallinity than para-aramid [15,29]. The difference in the arrangement of the structure of both aramids affects the availability of individual functional groups. Due to the hierarchical structure of para-aramid, the carbonyl, amino groups and the phenyl ring are better available compared to the meta-aramid, which is disordered, and the carbonyl and amino groups in the “crumpled” structure may hide between the phenyl ring and limit their availability and hence the lower oxygen content [15].

**Figure 5:**
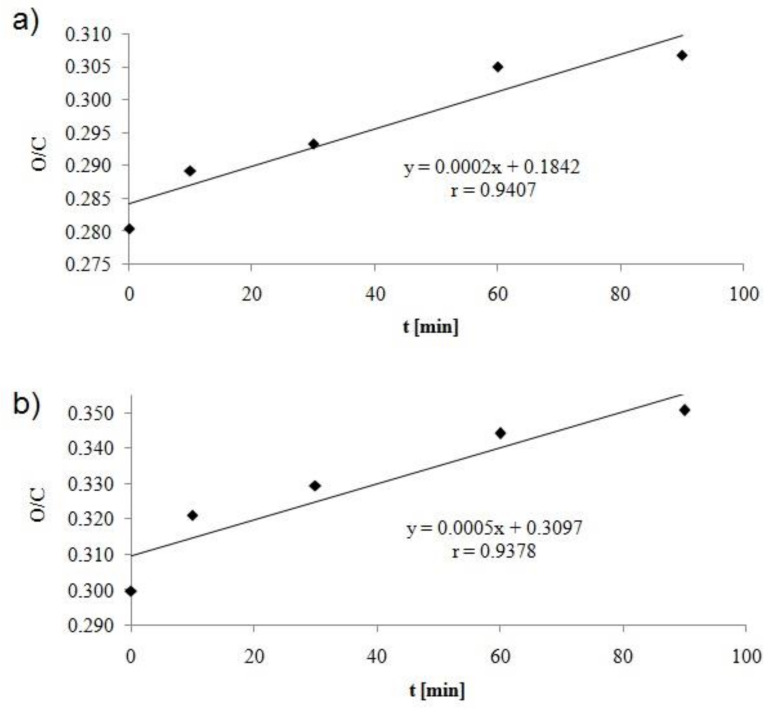
Influence of activation time on O/C value for (**a**) mAr and (**b**) pAr yarn.

Figure 5 shows the effect of activation time on changes in O/C ratio on the fiber surface. For both yarns, the O/C value increases linearly as the activation time increases, but they are higher for pAr yarns.For 90 min, the increase in the O/C ratio is 10% and 17% for mAr yarn and pAr, respectively. An increase in O/C was also observed in the earlier work of the authors after corona discharge modification of the meta- and para-aramid fabric [15].

An increase of O/C was found by Wu et al. [27], Wang et al. [16], Hwang et al. [20], and Jia et al. [28] for para-aramid fibers and Guo et al. [25] for para-aramid fabric. A decrease in the O/C value for para-aramid yarn was observed by Jia et al. [8], Inagaki et al. [29], and Chen et al. [24].

### 2.2. Goniometric Analysis

Table 3 summarizes the values of contact angle for yarns before and after activation in plasma for water (θ_W_), θformamide (θ_F_) and diiodomethane (θ_DIM_). The values of θ_W_, θ_F,_ and θ_DIM_ decrease with the increase of activation time. The value of θ_W_ form Ar/0 is 65.4 deg. For subsequent activation times it decreases by 16%, 30%, 35% after 10, 30 and 60 min, respectively. For mAr/90, the θ_W_ value stays at the same level as for mAr/60. The value of θ_F_ decreases by 4%, 17%, 24%, and 36% compared to 53.9 deg for mAr/0. The value of θ_DIM_ decreases by 16%, 20%, 30% after 10, 30, and 60 min compared to 60.2 deg for mAr/0. For mAr/90, the θ_DIM_ value does not change in comparison with mAr/60.

For pAr/0, the value of θ_W_ is 69.9 deg. After subsequent activation times, it decreases by 19%, 32%, 39% and 43%, respectively. The θ_F_ for pAr/0 amounts 64.2 deg and decreases by 10%, 20%, and 27% after 10, 30, and 90 min, respectively. After 60 min, the θ_F_ value is close to the value after 30 min. In turn, the θ_DIM_ value decreases respectively by 3%, 7%, 9%, and 20%, compared to the pAr/0 (56.9 deg).

Sheu et al. [22] and Wang et al. [16] obtained a similar value of water contact angle for para-aramid yarn. They noted higher decrease of its value after plasma treatment than we obtained. Wang et al. [16] found the higher decrease of the diiodomethane contact angle for para aramid fibers. Jia et al. [7] and Biswas et al. [9] obtained lower decrease for the water contact angle for para-aramid fibers and in contrast to us, no changes for diiodomethane. Ren et al. [30] found similar to us decrease of the water contact angle value for para-aramid fibers.

**Figure 6:**
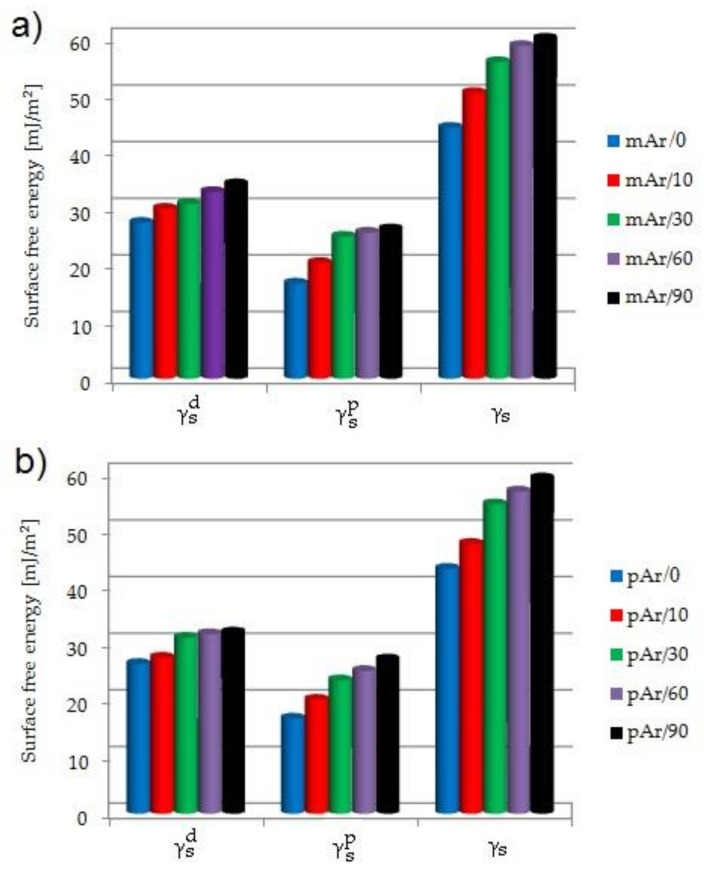
The surface free energy (γ_s_) of (**a**) mAr, (**b**) pAr yarn before and after plasma treatment (γ_s_^d^ - dispersive component, γ_s_^p^ - polar component), calculated from the average contact angle values.

For mAr/0, the γ_s_ value is 44.3 mJ/m^2^ (Figure 6a). After 10 min, the γ_s_ value increases by 13%. For subsequent times, the increase by 26%, 33%, and 37% is observed. Low-pressure RF plasma is generated in the residue air. In this condition, oxygen and water can be the source of ozone, atomic oxygen, and hydroxyl radicals that initiate the processes of oxidation and hydrolysis on the surface of fibers. The amide groups in the aramid chains are attacked, and the amide bond breaks. These phenomena, together with the generated high energy of UV radiation, opens the possibility of some crosslinking between the chains. After breaking the amide bonds in the chain of aramid fibers, radicals are formed that react with oxygen. Then the created peroxides decompose, which results in the formation of carboxylic acid and nitroso groups [26]. The formation of new functional groups on the surface of aramid fibers, e.g., hydroxyl, nitroso or carboxyl groups, make the fibers more hydrophilic, as evidenced by the decrease in water contact angle (Table 3) and an increase in the polar component value (γ_s_^p^) (Figure 6a), which for mAr/0 amounts 16.8 mJ/m^2^. A significant increase in the γ_s_^p^ value by 22% and 49% is observed especially after 10 and 30 min of activation, respectively. For longer times, the γ_s_^p^ value stays at the same level as for mAr/30. The dispersion component (γ_s_^d^) (Figure 6a) also increases with increasing activation time. For mAr/0, the γ_s_^d^ value is 27.5 mJ/m^2^. The γ_s_^d^ value increases by 9% after 10 min. For mAr/30, the γ_s_^d^ value does not change in comparison with mAr/10 and for mAr/60 and mAr/90, the γ_s_^d^ value increases by 20% and 25%, respectively. The increase in γ_s_^d^ value is associated with significant changes in the fiber surface topography (Figure 1, Figure 2). As the activation time increases, the surface roughness and unevenness increases, and the values of stereometric parameters increase, which indicates greater surface development.

Ji et al. [14], according to the Fowkes model, obtained the lower γ_s_ and γ_s_^p^ values for meta-aramid yarn and both values increased less than in our work, respectively by 10% and 15% after activation in the plasma.

The γ_s_ value of pAr/0 and mAr/0 are comparable (Figure 6b). For pAr/10, the γ_s_ value increases by 10% compared to pAr/0. After 30 min, a 26% increase in γ_s_ value is observed. For subsequent times a slight increase by4% and 8% for pAr/60 and pAr/90, respectively, are observed. As for mAr yarns, for pAr yarns the formation of new functional groups on the surface of the fibers, e.g., hydroxyl, nitroso or carboxyl groups, has led to an increase in the hydrophilic nature of the fibers, as evidenced by the decrease in the water contact angle (Table 3) and an increase in the component polar (γ_s_^p^) (Figure 6b). The γ_s_^p^ value of pAr/0 is 16.8 mJ/m^2^. After subsequent activation times, an increase of 20%, 40%, 50%, and 62%, respectively, is observed. The dispersion component (γ_s_^d^) (Figure 6b) also increases with increasing activation time. For pAr/0, the γ_s_^d^ amounts 26.4 mJ/m^2^. After 10 min of activation, a slight increase of 4% is observed. A much larger increase by 18% is found for pAr/30. For subsequent times the γ_s_^d^ value stays at the level similar to pAr/30. Wang et al. [16], according to the Owens-Wendt formula, found the higher γ_s_ value and lower γ_s_^p^ and γ_s_^d^ values for para-aramid yarn. Similarly to us, after activation, they observed an increase in the γ_s_ and γ_s_^p^ values, and in contrary to us, the decrease in γ_s_^d^ value. Jia et al. [7] observed the higher γ_s_ and γ_s_^d^ values and lower γ_s_^p^ value for para-aramid fibers than we obtained. After activation, they found an increase of the γ_s_ and γ_s_^p^ value and a decrease of the γ_s_^d^ value (calculated according to the Owens-Wendt formula).

### 2.3. FTIR Analysis

Table 4 summarizes the characteristic bands and their wavenumbers for mAr/0 and pAr/0 yarn [31,32,33].

**Figure 7:**
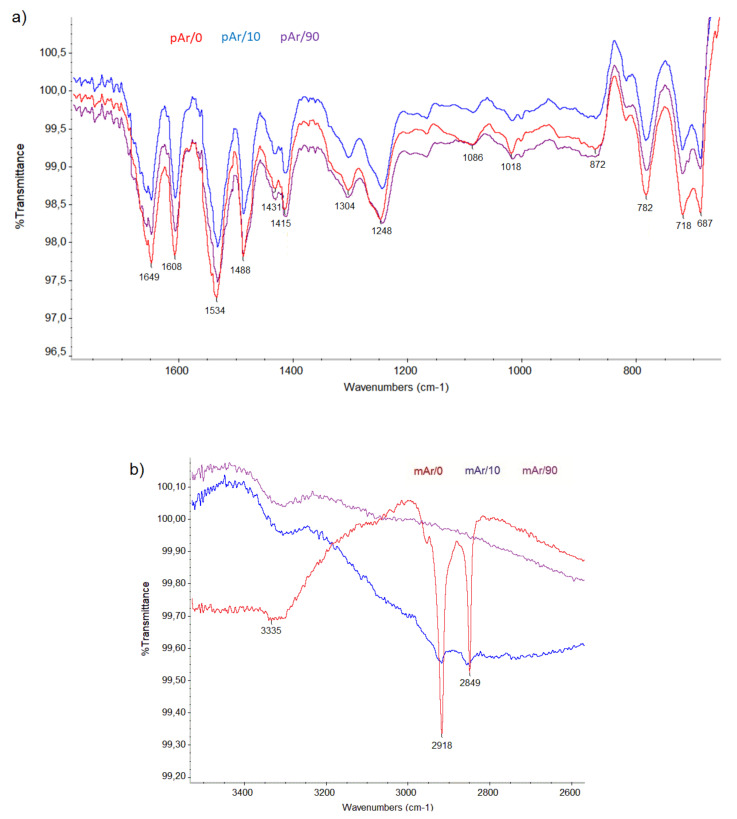
The FTIR spectra of unmodified and plasma-treated mAr yarns of: (**a**) 600–1800 cm^−1^, (**b**) 2600–3550 cm^−1^.

**Figure 8:**
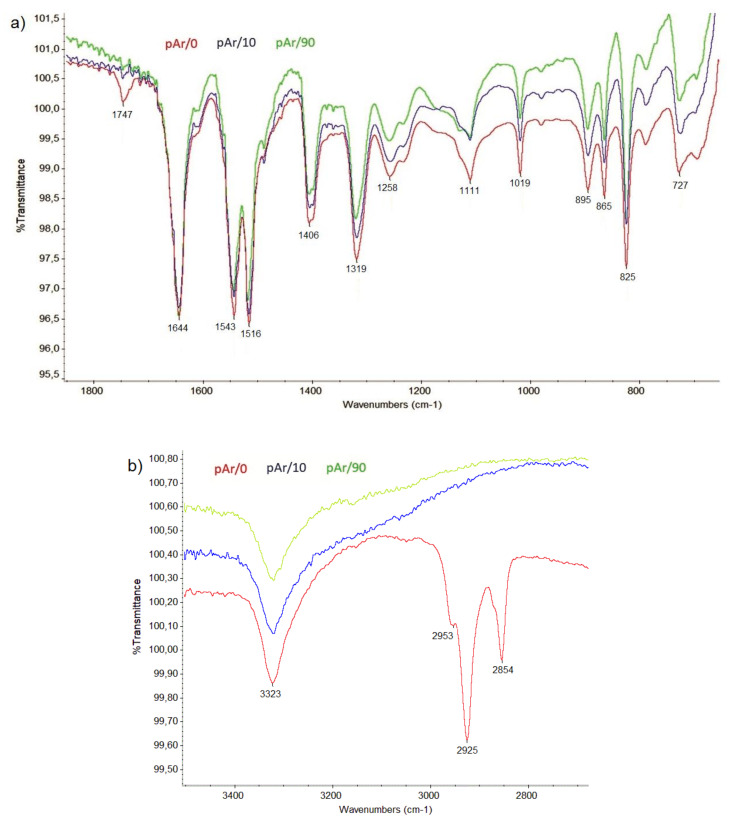
The FTIR spectra of unmodified and plasma-treated pAr yarns of: (**a**) 650–1850 cm^−1^, (**b**) 2700–3500 cm^−1^.

In the spectra for both yarns (Figure 7, Figure 8), a double band at 2849 and 2918 cm^−1^ for mAr/0 and 2854 and 2925 cm^−1^ for pAr/0 is observed. These bands indicate the presence of alkyl chain what indicate the presence of finishing agent on the surface of the fibers. For pAr also carbonyl bound (1748 cm^−1^) is present as a structural fragment of mention finishing agents that are removed during plasma treatment during the first 10 min of activation. Because changes in surface topography can be observed without their influence on the FTIR spectrum, in this situation it can be concluded that during plasma treatment, aramids break down into low molecular weight fragments that are removed from the surface of the fibers. Low pressure in the plasma chamber favors the removal process of small molecular weight products of fibers surface etching.

### 2.4. DSC Analysis

**Figure 9:**
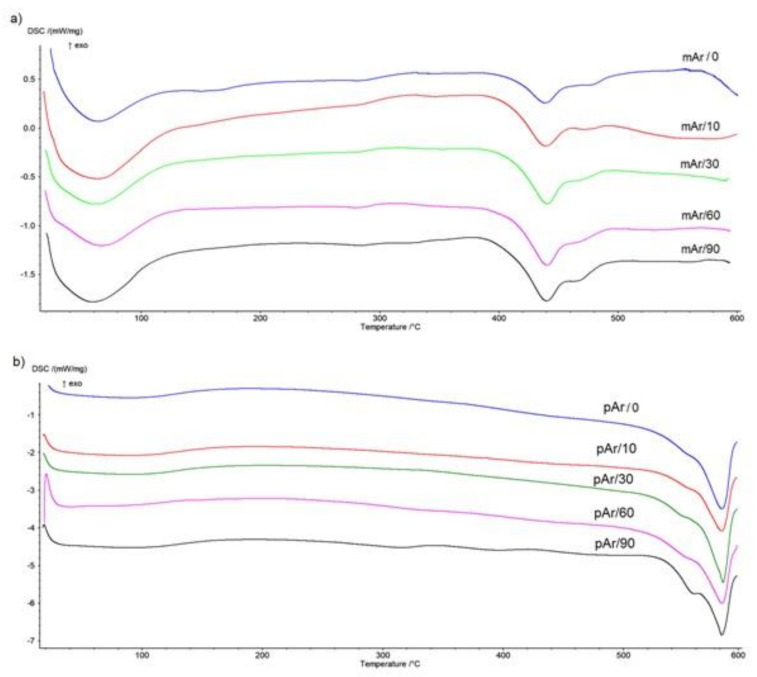
DSC thermograms of thermal decomposition process of the unmodified and plasma-treated (**a**) mAr, (**b**) pAr yarns.

On the DSC curve for the mAr/0 (Figure 9a), there is an endothermic peak in the range 20–120 °C, which indicates water desorption. The degradation process occurs in two stages in the range of 406–491 °C, with maximaof endothermic peaks at 439 °C (first stage) and 478 °C (second stage), and the heat of thermal decomposition is 95 J/g (Table 5). As the activation time increases, the initial temperature of thermal degradation increases by 4 °C, 6 °C, 8 °C, and 12 °C, for mAr/10, mAr/30, mAr/60 and mAr/90, respectively. The final and in the peak 1 maximum temperature does not change significantly, and the temperature in peak 2 maximum decreases with an increase of activation time by 4 °C, 6 °C, 10 °C, and 12 °C, respectively. The heat of thermal decomposition increases by 6%, 11%, 22%, and 50%, respectively.

In the case of pAr/0 (Figure 9b), we also observe an endothermic peak in the range of 20–120 °C. The pAr/0 degradation process occurs in two stages in the range of 525–591 °C, with endothermic peaks maximum at 552 °C (first stage) and 582 °C (second stage), and the heat of thermal decomposition amounts 271 J/g. The initial temperature of thermal degradation of yarns increases with the increase of activation time by 3 °C, 5 °C, 7 °C, and 10 °C for pAr/10, pAr/30, pAr/60, and pAr/90, respectively. The final temperature does not change. The temperature of peak 1 for yarn pAr/90 increased by 6 °C compared to yarn pAr/0. For shorter activation times, it was slightly lower compared to yarn pAr/0. The temperature at the peak 2 maximum stays at the same level for all activation times. After 10 min of activation, the heat of thermal decomposition has not changed significantly. A significant increase in ΔH_Deg_ by 14% is observed for pAr/30. The heat of thermal decomposition for two subsequent times is 2% and 6% higher, compared to pAr/30.

An increase in the value of heat of thermal degradation after modification of meta- and para-aramid fabric through corona discharge was observed in our previous work [15].

### 2.5. TG/DTG Analysis

**Figure 10:**
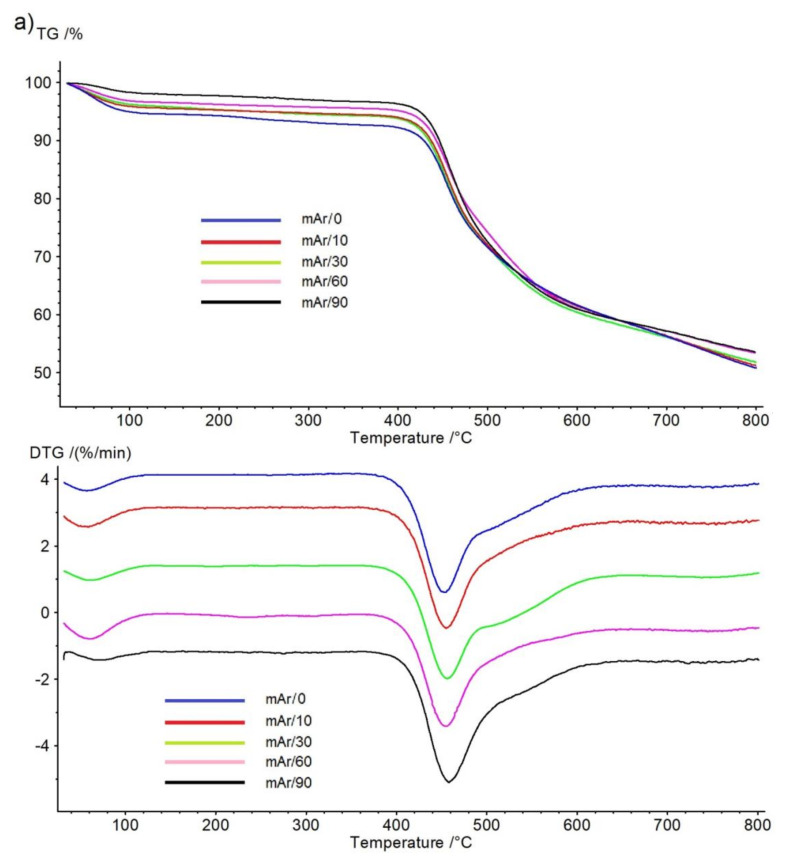

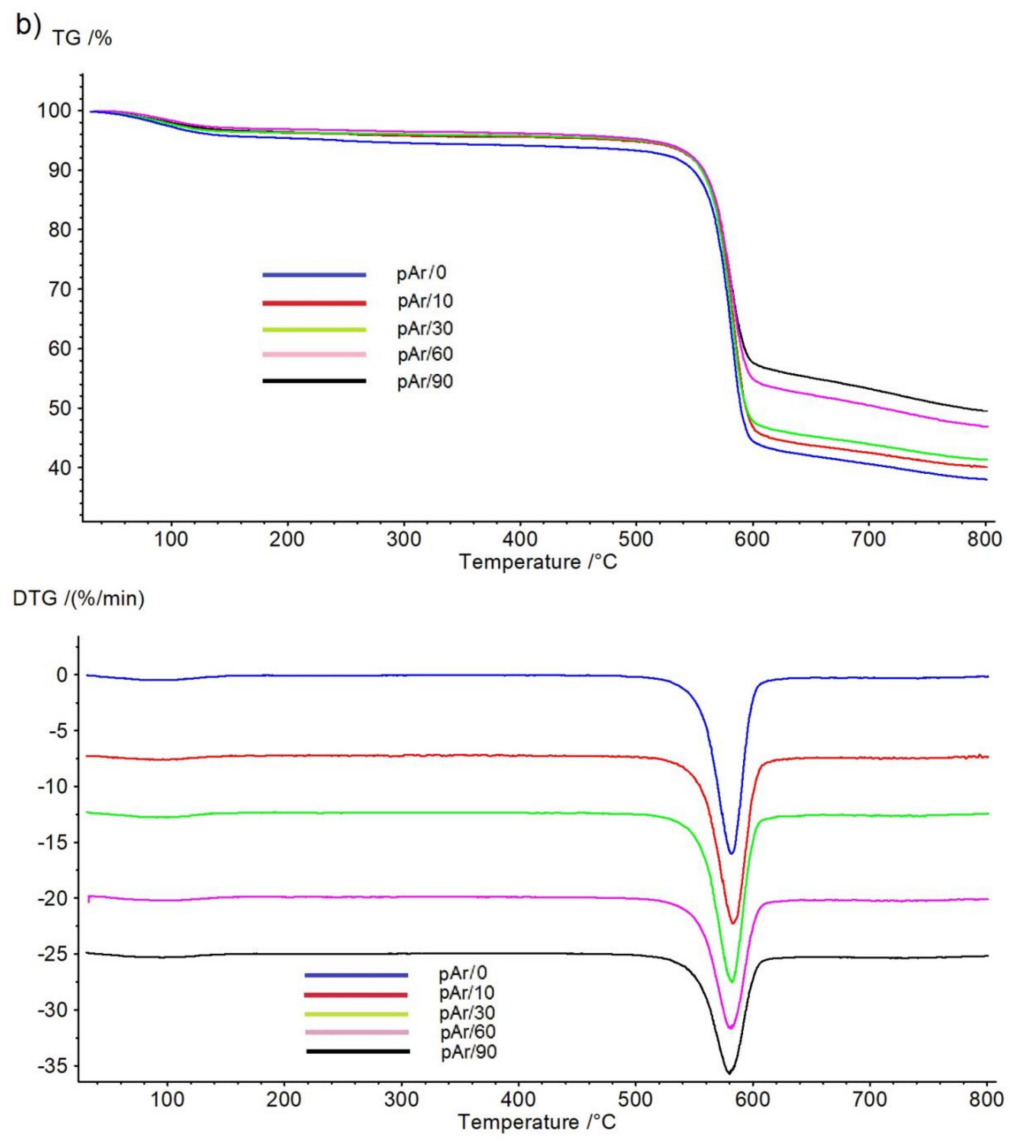
TG/DTG thermograms of thermal decomposition process of the unmodified and plasma-treated (**a**) mAr, (**b**) pAr yarns.

On the TG curve for mAr/0 yarn (Figure 10a) a slight weight loss is observed in the range of 30–120 °C, which corresponds to the water desorption peak on the DTG curve. The thermal decomposition process occurs in two stages in the temperature range of 414–583 °C with a maxima at 455 °C and 521 °C for peak 1 and peak 2, respectively (Table 6). Weight loss at 800 °C is 49%. Activation of the yarn does not cause significant changes in the initial temperatures, and the final temperature increases by 9 °C, 13 °C, 19 °C, and 21 °C after 10, 30, 60, and 90 min of activation, respectively. The temperature in the peak 1 maximum for yarn mAr/10, mAr/30, mAr/60 stays at the same level, and for yarn mAr/90, there is a temperature increase in peak 1 by 3 °C. The temperature at the maximum of peak 2 increases by 4 °C for mAr/10, similar to mAr/30. For mAr/60 and mAr/90, an increase in temperature of 9 °C and 21 °C is observed. Weight loss decreases by 0.4%, 1%, 3%, and 3% after 10, 30, 60, and 90 min of activation, respectively.

For pAr/0 yarn, the TG curve (Figure 10b) shows a weight loss in the range of 30–120 °C, which corresponds to the water desorption peak on the DTG curve. The thermal decomposition process takes place in one-step at a temperature range of 560–599 °C with a maximum at 581 °C. The weight loss of pAr/0 at 800 °C is 62%. An increase in the activation time causes a slight increase in the value of the initial temperatures. After 90 min, the initial temperature increases by 4 °C. The final and maximum peak temperatures do not change with increasing activation time. Weight loss decreases by 3%, 5%, 14%, and 19%.

Sun et al. [35] also observed a reduction in a weight loss of para-aramid fibers by about 2% after activation in an oxygen plasma (300 V). In our previous work [15], we also found a decrease in weight loss for meta- and para-aramid fabric.

Differences in thermal properties and changes as a result of low-pressure air RF plasma modification are associated with differences in the production method and physicochemical properties of both fibers. The mAr fiber is made by spinning in a chemical solution called the wet spinning method. This semi-crystalline fiber is made up of a molecular chain partially oriented along the fiber axis. Its average moisture recovery is 4.5% in a standard atmosphere [15,36,37,38,39].

The pAr fibers are formed by the dry-jet, wet spinning method. The presence of an amide bond in the polymer chain promotes the formation of a long chain, helping to form liquid crystals. The pAr fiber consists of fully stretched liquid crystal chains formed along the fiber axis with a very high degree of crystallinity and very high orientation. In a normal atmosphere, its average moisture recovery is about 4.3%. Due to the difference in the degree of crystallinity, the pAr density is 1.44 g/cm^3^, and mAr 1.38 g/cm^3^ [15,39].

Oxidation by air plasma treatment causes changes on the surface of the aramid fibers, without significantly affecting the thermal properties and cause slight changes in degradation temperatures. The used treatment time influences on the water content of both yarns (Figure 7a,b). We observe a decrease in water weight loss from about 6% to about 2% for mAr yarns and from about 5% to about 3% for pAr yarns.

### 2.6. Mechanical Properties

**Figure 11:**
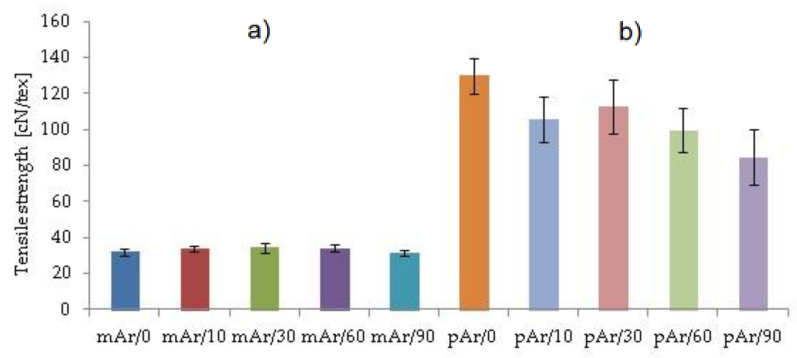
The specific strength of the unmodified and plasma-treated (**a**) mAr, (**b**) pAr yarns.

The specific strength of the mAr/0 yarn is 32.1 cN/tex (Figure 11) and is 4 times smaller than the pAr/0 yarn (129.9 cN/tex). To verify that changes in the specific strength values after modification in the plasma are statistically significant, an analysis of significant differences is performed using the t-Student’s test. Experimental t values (Table 7) are calculated for p = 0.05 and t_(0.05, 10)_ = 2.10 (t values are taken from statistical tables) for average specific strength values.

For mAr yarns after activation, the specific strength value slightly increases by 6%, 7%, and 7%, after 10, 30, and 60 min activation, respectively. After 90 min decreases by 2%. A statistically significant increase is observed after 30 and 60 min of activation (Table 7). For pAr yarns, the specific strength value decreases by 19%, 13%, 23%, and 35% after 10, 30, 60, and 90 min, respectively. These changes are statistically significant for all activation times. A slight increase in the strength of mAr yarns and a decrease in strength of pAr yarnsafter plasma treatment is caused by the difference in the crystallinity of both types of aramids. The disordered mAr/0 structure and a much lower degree of crystallinity mean that the fibers of mAr/0 yarn are less susceptible to plasma, so the chains can change their position in the structure and become more durable. In turn, the ordered and high-crystalline pAr/0 causes that it undergoes more damage caused by the action of the plasma. Its structure is very stiff and the chains cannot freely change their position in the structure of fibers under the influence of plasma.

Jia et al. [36] also found the para-aramid fibers tensile strength decrease after activation. A slight decrease in the breaking strength of para-aramid fibers observed Biswas et al. [9]. In contrary to us, Liu et al. [37] did not observe significant changes in the tensile strength of para-aramid yarn after plasma modification. In turn, Inagaki et al. [29] and Hwang et al. [20] registered a slight increase in tensile strength for para-aramid yarn.

## 3. Materials and Methods

### 3.1. Materials

**Figure 12:**
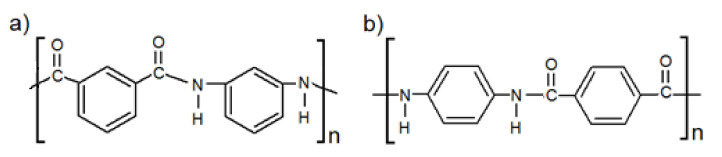
Chemical formulas of: (**a**) meta-, (**b**) para-aramid yarn.

**Figure 13:**
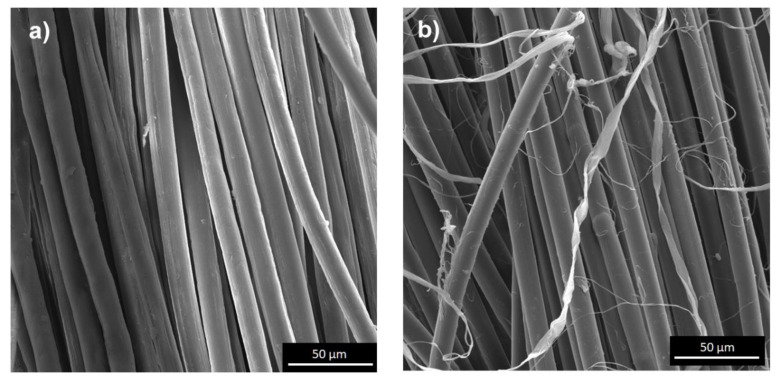
SEM images of (**a**) mAr and (**b**) pAr yarn (1000× magnification).

Two doubled aramid staple yarns; meta-aramid (mAr), poly(isophthalates-1,3-fenylodiamid) (Figure 12a), NewStar^®^ (Yantai Tayho Advanced Materials Co., Ltd.,Yantai, China) and para-aramid (pAr), poly(1,4-terephthalate-fenylodiamid) (Figure 12b), Kevlar^®^ (DuPont, London, United Kingdom) were studied (Figure 13, Table 8).

### 3.2. Low-PressureAir RF Plasma Treatment

The meta- and para-aramid yarns were washed in diethyl ether for 30 min and dried in 25 °C. After dissection yarns were activated in low-pressure air RF (40 kHz) plasma (Diener, Zepto, Ebhausen, Germany) for 10, 30, 60, and 90 min. Pressure in the plasma chamber was set up at 0.3 mbar level with the air as a working gas. The power of plasma was set up on a maximum level of 50 W.

### 3.3. Instrumental Techniques

The SEM/EDS analysis of yarns was performed using a scanning electron microscope (SEM) VEGA 3 (TESCAN, Brno, Czech Republic) with an Energy Dispersive Spectroscopy (EDS) X-ray microanalyzer INCA Energy (Oxford Instruments Analytical, Halifax, United Kingdom) with a magnification of 1000× and 20000×. Samples of textile materials were placed on the platform (diameter 12 mm) and fixed with an adhesive carbon disc. Three X-ray spectra were recorded for each sample and mean values of the percentage of elements were determined. The size of the analyzed surfaces of samples was 0.05 mm^2^. The 3D SEM images of yarns were taken using Alicona MeX software. Based on the 3D SEM analysis, the values of an average height of selected area (S_a_), the root-mean-square height of selected area (S_q_), the maximum peak height of selected area (S_p_), the maximum valley depth of selected area (S_v_), the maximum height of selected area (S_z_), the root mean square gradient (S_dq_), developed interfacial area ratio (S_dr_) were determined.

The analysis of the surface properties was carried out by the goniometric method using goniometer DSA100 (Kruss, Hamburg, Germany). To determine the surface free energy (γ_s_), three standard liquids with known surface tensions and different values of dispersive and polar components were applied (Table 9). The drop of liquid with a volume of 4 µL was applied. Three repetitions for each sample were used. Surface free energy was determined according to the Wu model (Equation 1) [38,39,40,41] which based on the assumptions of the Owens-Wendt model but describes the intermolecular interaction using the harmonic mean.

γ_l_(1+cosθ) = 4((γ_l_^d^γ_s_^d^)/ γ_l_^d^ + γ_s_^d^ + γ_l_^p^γ_s_^p^/γ_l_^p^ + γ_s_^p^) and γ_s_ = γ_s_^d^+ γ_s_^p^ (1)

where γ_s_^d^, γ_s_^p^ are the dispersion and polar components of the solid, γ_l_^d^, γ_l_^p^ are the dispersion and polar components of the liquid.

FTIR/ATR (Fourier Transform Infrared Spectroscopy/Attenuated Total Reflectance) spectra of reference and plasma-treated yarns were recorded in the range 600–4000 cm^−1^ using Nicolet IS 50 spectrometer (Thermo Fisher Scientific Inc., Bartlesville, OK, USA) with GATR (grazing angle attenuated total reflectance) accessory (Harrick Scientific Products, Inc., New York, NY, USA) using the MCT (Mercury-Cadmium-Telluride)detector. This accessory allows to collecting spectra from the surface of the fibers at a depth of about up to 50 nm.

Investigations of thermal properties of reference and plasma-treated yarns were carried out using the differential scanning calorimeter DSC 204 F1 Phoenix (Netzsch, Selb, Germany). Yarns with a weight of about 5 mg were placed in aceramic cruciblewith a volume of 85 μL and heated in the temperature range 20–600 °C with a rate of 10°C/min under nitrogen (gas flow 20 mL/min). Three samples of each tested yarns were studied. The initial (T_Onset_), final (T_End_), and the peaks maxima (T_Peak1_, T_Peak2_) temperature and the heat of thermal decomposition (ΔH_Deg_) were determined.

Thermogravimetric analysis TG/DTG was performed using thermogravimetric analyzer TG 209 F1 Libra (Netzsch, Selb, Germany). Samples of yarns with a mass of 5 mg were tested in a ceramic crucible with a volume of 85 μl in a nitrogen atmosphere (gas flow of 25 mL/min.). Yarns were heated in the temperature range was 30–800 °C with aheating rate 10 °C/min. Three repetitions were used. The initial (T_Onset_), final (T_End_), and the peaks maxima (T_Peak1_, T_Peak2_) temperature of the thermal degradation process and the weight loss of samples at 800 °C were determined.

The study of mechanical strength of 15 cm long reference and plasma-treated yarns was tested using Instron 3367 Test Machine (Instron, High Wycombe, United Kingdom) in accordance with PN-EN ISO 2062: 2010 “Textiles - yarns from packages - determination of single-end breaking force and elongation at break”. Ten repetitions for each sample were used.

## 4. Conclusions

The purpose of the research was to change the surface free energy and its polar and dispersive component of meta- and para-aramid yarns usinglow-pressure air RF plasma the impact of process parameters on changes in thermal and mechanical properties. Changes in yarns properties depend on the activation time and type of aramid.SEM analysis of both aramid fibers surface showed changes in topography and an increase in its unevenness and roughness after subsequentplasma activation times. The contact angles for water, formamide, and diiodomethane decreased with increasing activation time. The yarns have become more hydrophilic. The value of the free surface energy of both yarns is comparable and amounts to about 44 mJ/m^2^. For meta-aramid yarn after 10 and 90 min of activation, the SFE value increased respectively by 13.8% and 37.0%, whereby the polar component increased from 16.8 mJm^2^ by 21.8% and 57.1% and the dispersion component increased from 27.5 mJ/m^2^ by 9.0% and 24.8%. In the case of para-aramid yarn for these treatment times, the SFE value increased by 10.2% and 37.0%. The polar component increased from 16.8 mJ/m^2^ by 19.9% and 62.3% and the dispersion component increased from 26.4 mJ/m^2^ by 4.1% and 21.0%. For both types of yarns, the value of the polar component increased to a greater extent than the dispersion component. The oxygen content also increased for both yarns, while carbon and nitrogen decreased slightly with increasing activation time. FTIR analysis showed that plasma treatment caused the removal of the finishing agent from the surface, which originated from the yarn manufacturing process. The thermal properties of both yarns have slightly improved. DSC analysis showed that the initial thermal decomposition temperature increased for both aramid yarns. The heat of thermal degradation amounted 95 J/g and 271 J/g for meta- and para-aramid yarn, respectively. After 10 to 90 min of activation, it increased by about 6–50% for meta-aramid yarn. For para-aramid yarn, it didnot change after 10 min and increased after 90 min by about 21%.

TG analysis showed that the weight loss amounted 49% and 62% for meta- and para-aramid yarn. After 10 min and 90 min treatment values decreased respectively from 0.4% to 3% for meta-aramid yarn and from 3% to 19% for para-aramid yarn. Shifting temperatures towards higher values and increasing heat of thermal degradation and reducing weight loss indicates that aramids are becoming more thermally stable, which is very important for further functionalization.

After 10 min of activation, the specific strength of meta-aramid yarn increased from 32 cN/tex by 6% and after 90 min, it decreased by 2%. For para-aramid yarn, the specific strength value decreased from 130 cN/tex by 19% and 35%, respectively.

Compared to our work, the differences in the chemical composition of the fiber surface, changes in surface topography, their thermal resistance, or strength, observed by other authors, may result from differences in the etching of the surface of the oxidized fiber due to the use of different types of plasmas and yarns/fabric. Therefore, conditions of plasma processing and the structure of the modified textile materials have a significant impact on the results obtained.

The low-pressure air RF plasma and modification parameters were used for the first time. It gives an advantage over other types of plasma because it changes the surface of aramid fiber sand causes weakness of the para-aramid yarn and no significant changes in the strength of the meta-aramid yarn. Low-pressure air RF plasma causes an increase in free surface energy, resulting from a greater increase in the polar component associated with dipoles generated on the surface of the fibers, and less from Van der Waals forces associated with a slight increase in the dispersion component for para-aramid yarn, and a slight increase in polar component and a greater increase in dispersive component for meta-aramid yarn.

For both types of yarn, 10 min of plasma activation is sufficient to prepare their surface for the planned nanomodification, because of the increase of specific strength for meta-aramid yarn and its lowest decrease for para-aramid yarn. Moreover, changes in surface topography and increase in hydrophilicity are sufficient for the lowest time of activation. Choosing the appropriate parameters of plasma activation is an important part of the research, allowing the use of modifiers on the surface of aramid textile materials to give them new, multifunctional properties, e.g., conductive, bioactive, resistant to UV radiation, etc.

## Figures and Tables

**Table 1:** The mean values of the weight percentages of elements with standard deviations for reference and treated mAr and pAr yarns.

	Weight Percentages of Elements, wt.%				
Sample	C	N	O	Na	S
mAr/0	68.0 ± 0.3	12.9 ± 0.1	19.1 ± 0.2	0.1 ± 0.0	
mAr/10	67.0 ± 0.8	13.7 ± 0.7	19.4 ± 0.2	0.1 ± 0.0	
mAr/30	67.3 ± 0.5	12.8 ± 0.7	19.7 ± 0.2	0.1 ± 0.0	
mAr/60	67.1 ± 0.7	12.4 ± 0.7	20.5 ± 0.3		
mAr/90	67.2 ± 0.5	12.2 ± 0.7	20.6 ± 0.3		
pAr/0	67.7 ± 0.6	11.0 ± 0.7	20.3 ± 0.4	0.5 ± 0.0	0.5 ± 0.1
pAr/10	66.4 ± 0.7	11.3 ± 0.7	21.4 ± 0.3	0.6 ± 0.0	0.5 ± 0.1
pAr/30	66.1 ± 0.7	11.0 ± 0.7	21.8 ± 0.1	0.6 ± 0.1	0.5 ± 0.1
pAr/60	65.3 ± 0.7	10.7 ± 0.6	22.5 ± 0.2	0.8 ± 0.0	0.8 ± 0.0
mAr/90	65.8 ± 0.3	10.0 ± 0.3	23.1 ± 0.2	0.6 ± 0.0	0.6 ± 0.0

**Table 2:** Analysis of statistical significance (t) of changes in elements content based on t-Student test for t_(0.05, 3)_=2.78 ( t values taken from statistical tables).

		Experimental t Value		
Sample		t_C_	t_N_	t_O_
mAr/0 vs	mAr/10	2.07	2.10	1.68
	mAr/30	0.12	0.01	0.23
	mAr/60	1.96	1.10	6.14
	mAr/90	0.53	2.60	5.68
pAr/0 vs	pAr/10	2.56	0.42	3.73
	pAr/30	3.03	0.04	6.32
	pAr/60	4.71	0.59	8.76
	pAr/90	1.33	2.96	9.80

**Table 3:** The mean values of water (θ_W_), formamide (θ_F_) and diiodomethane (θ_DIM_) contact angle and its standard deviation for meta- and para-aramid yarns.

Sample	Contact Angle		
	Θ_W_, deg	Θ_F_, deg	Θ_DIM_, deg
mAr/0	65 ± 4	54 ± 6	60 ± 5
mAr/10	55 ± 4	52 ± 5	50 ± 4
mAr/30	46 ± 1	45 ± 4	48 ± 7
mAr/60	43 ± 6	41 ± 5	42 ± 7
mAr/90	41 ± 4	35 ± 8	41 ± 6
pAr/0	70 ± 3	64 ± 7	57 ± 5
pAr/10	57 ± 3	57 ± 6	55 ± 6
pAr/30	47 ± 2	52 ± 4	53 ± 3
pAr/60	43 ± 6	50 ± 6	52 ± 8
mAr/90	40 ± 3	46 ± 6	46 ± 5

The values of the free surface energy (γ_s_) SFE of mAr/0 and pAr/0 yarns increase with the treatment time.

**Table 4:** FTIR bands assignments for mAr and pAr yarn.

mArYarn		pArYarn	
Bands (cm^−1^)	Description [31,32,33]	Bands (cm^−1^)	Description [7,10,34]
687	C-H out-of-plane in meta substituted aromatic ring	821	p-substituted phenyl
718	N-H out-of-plane bending	1306	C-N stretching
782	C-H out-of-plane in meta substituted aromatic ring	1509	C=C stretching
1248	C-N stretching, N-H in-plane bending, C-C stretching (Amide III)	1538	N-H bending
1304	Aromatic C-N stretching	1637	C=O stretching
1534	N-H in-plane bending, C-N stretching coupled modes of the C-N-H group	1740	C=O stretching
1608	C=C stretching vibrations of anaromatic ring	3323	O-H and N-H stretching vibrations
1649	C=O stretching (carbonyl group), C-N stretching		
3335	O-H and N-H stretching vibrations		

**Table 5:** DSC parameters of unmodified and plasma treated mArand pAryarns.

Sample	T_Onset_ [^o^C]	T_End_ [^o^C]	T_Peak1_ [^o^C]	T_Peak2_ [^o^C]	ΔH_Deg_ [J/g]
mAr/0	406 ± 2 *	491 ± 0	439 ± 0	478 ± 0	95 ± 2
mAr/10	410 ± 1	493 ± 0	439 ± 1	474 ± 0	100 ± 1
mAr/30	412 ± 1	492 ± 0	439 ± 1	472 ± 0	106 ± 1
mAr/60	414 ± 1	491 ± 0	440 ± 0	467 ± 1	116 ± 3
mAr/90	418 ± 1	492 ± 0	440 ± 0	466 ± 0	143 ± 5
pAr/0	527± 1	591 ± 0	552 ± 0	582 ± 1	271 ± 2
pAr/10	530 ± 1	590 ± 0	549 ± 0	583 ± 1	272 ± 2
pAr/30	532 ± 1	591 ± 0	550 ± 1	581 ± 1	310 ± 4
pAr/60	534 ± 1	590 ± 0	551 ± 0	582 ± 1	315 ± 5
pAr/90	537 ± 1	590 ± 0	558 ± 0	582 ± 1	329 ± 4

* average value ± standard deviation.

**Table 6:** Thermogravimetry/Derivative Thermogravimetry (TG/DTG) parameters of unmodified and plasma-treated mAr and pAr yarns.

Sample	T_Onset_ [°C]	T_End_ [°C]	T_Peak1_ [°C]	T_Peak2_ [°C]	Weight Loss 800 °C [%]
mAr/0	414 ± 1 *	583 ± 0	455 ± 0	521 ± 1	49 ± 0
mAr/10	414 ± 1	592 ± 1	455 ± 1	525 ±0	49 ± 0
mAr/30	414 ± 1	596 ± 1	454 ± 0	525 ± 0	48 ± 0
mAr/60	413 ± 1	602 ± 1	456 ± 0	530 ± 1	47 ± 1
mAr/90	415± 1	604 ± 1	458 ± 0	542 ± 0	46 ± 1
pAr/0	560 ± 1	599 ± 1	582 ± 0		62 ± 1
pAr/10	561 ± 1	603 ± 0	583 ± 1		60 ± 1
pAr/30	562 ± 1	598 ± 1	582 ± 1		59 ± 1
pAr/60	564 ± 0	600 ± 1	581 ± 1		53 ± 1
pAr/90	564 ± 1	600 ± 1	580 ± 1		50 ± 1

* average value ± standard deviation.

**Table 7:** Analysis of statistical significance (t) of changes in elements content based on t-Student test for t_(0.05, 10)_=2.10 ( t values taken from statistical tables).

Experimental t Value			
mAr/0 vs mAr/10	mAr/0 vs mAr/30	mAr/0 vs mAr/60	mAr/0 vs mAr/90
2.00	2.28	2.36	0.47
pAr/0 vs pAr/10	pAr/0 vs pAr/30	pAr/0 vs pAr/60	pAr/0 vs pAr/90
4.48	2.10	5.73	2.33

**Table 8:** Characteristic of aramid yarns.

Scheme	Parameters	mAr	pAr
	Fibers thickness (µm)	12.6 ± 0.7	12.4 ± 0.7
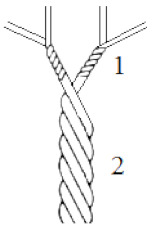	Linear density (tex)	25 × 2	20 × 2
	Number of twists [twist/m] and twist direction for single yarn- 1	574 ± 16 (Z)	477 ± 16 (S)
	Number of twists [twist/m] and twist direction for double yarn- 2	504 ± 13 (S)	548 ± 15 (Z)


**Table 9:** Characteristic of standard liquids.

	Surface Tension, mJ/m^2^		
Standard Liquid	γ_l_	γ_l_^d^	γ_l_^p^
Water (distilled)	72.8	21.8	51.0
Formamide (99.5%, Sigma-Aldrich)	58.0	39.0	19.0
Diiodomethane (99%, Sigma-Aldrich)	50.8	48.5	2.3

γ_l-_ surface free energy, γ_l_^d^-dispersive component, γ_l_^p^-polar component.
